# A Study on Cancer-Associated Fibroblast Using Alpha-Smooth Muscle Actin Immunohistochemistry in Oral Squamous Cell Carcinoma

**DOI:** 10.7759/cureus.85027

**Published:** 2025-05-29

**Authors:** Ambika Kunhikannan, T.N. Suresh, S.M. Azeem Mohiyuddin

**Affiliations:** 1 Pathology, Sri Devaraj Urs Medical College, Kolar, IND; 2 Pathology and Laboratory Medicine, Sri Devaraj Urs Medical College, Kolar, IND; 3 Otorhinolaryngology and Head and Neck Surgery, Sri Devaraj Urs Medical College, Kolar, IND

**Keywords:** alpha-smooth muscle actin, cancer-associated fibroblasts, lymph node metastasis, oral squamous cell carcinoma, tumor budding, tumor microenvironment, tumor-stroma ratio

## Abstract

Background

Oral squamous cell carcinoma (OSCC) represents a significant global health burden with complex pathophysiology involving tumor microenvironment interactions. The tumor stroma, particularly cancer-associated fibroblasts (CAFs), plays a crucial role in tumor advancement, invasion, and metastasis. CAFs, identified by alpha-smooth muscle actin (α-SMA) expression, influence tumor behavior through extracellular matrix remodelling and pro-tumorigenic signalling. Despite emerging evidence of their prognostic significance, the relationship between CAF expression patterns and clinicopathological parameters in OSCC remains inadequately characterized.

Objectives

This study aimed to detect CAFs using α-SMA immunohistochemistry in OSCC and evaluate their association with LNM and pTNM staging, potentially identifying new prognostic markers for clinical management.

Methodology

This laboratory-based analytical study was conducted between September 2022 and December 2023. Histopathologically confirmed OSCC cases (n=88) treated by composite resection and cervical lymph node dissection were included, excluding recurrent cases, patients who received neoadjuvant chemotherapy, and second primary cancers. H&E slides were reviewed for LNM and pTNM staging. Immunohistochemical staining for α-SMA was performed on 4 μm formalin-fixed paraffin-embedded tissue sections using heat-induced antigen retrieval, followed by incubation with primary antibody and horseradish peroxidase (HRP)-conjugated secondary antibody. CAF expression was quantified using Kellermann et al.'s scoring system (Score 1 is <1%, Score 2 is between 1% and 50%, and Score 3 is >50% stained cells) and categorized by distribution pattern (focal, network, or spindle). Additional parameters assessed included tumor-stroma ratio (TSR), worst pattern of invasion (WPOI), tumor budding (TB), and tumor-infiltrating lymphocytes (TILs).

Results

The study population comprised 88 patients (69.3% female, 30.7% male) (average age: 56.97 years). The buccal mucosa represented the most frequent site (50%), with well-differentiated tumors predominating (80.7%). Pathological staging revealed Stage IVA as the most prevalent (33%), followed by Stage III (31.8%). Regarding CAF expression, Score 3 (abundant CAFs) was observed in 55.7% of cases, Score 2 in 40.9%, and Score 1 in only 3.4%. Network pattern CAF distribution predominated (38.6%), with equal representation of focal and spindle configurations (30.7% each). Statistical analysis revealed no significant association between CAF scores and LNM (p=0.758); however, CAF distribution patterns demonstrated a statistically significant association with pTNM staging (χ²=26.716; p=0.001), with advanced stages showing a distinct pattern shift toward network and spindle arrangements. Notably, significant correlations were observed between TSR and CAF score (p<0.0001), TB and CAF score (p=0.021), and TB and LNM (p=0.047). More aggressive invasion patterns demonstrated higher CAF scores and increased TB intensity.

Conclusion

While CAF scores alone did not predict LNM, CAF architectural patterns demonstrated significant associations with pathological staging in OSCC. The correlations between CAF expression, TB, and invasion patterns suggest that CAF distribution may serve as a valuable prognostic indicator. These findings highlight the potential of CAF architectural evaluation as an adjunctive histopathological parameter for risk stratification in OSCC patients.

## Introduction

Oral squamous cell carcinoma (OSCC) signifies a significant global health problem, being the eighth most widespread cancer worldwide according to GLOBOCAN 2022 estimates, with approximately 500,000 new cases diagnosed annually [1]. In India, OSCC accounts for nearly 30% of all cancers, with an age-standardized incidence rate of 12.6 per lakh population, which is significantly higher than the global average of 4.1 per lakh [2,3]. The southern states of India, including Karnataka, report particularly concerning statistics, with OSCC accounting for up to 40% of all malignancies in some regional cancer centers [2]. In Karnataka specifically, oral cancer ranks as the most prevalent cancer among men and the third most prevalent cancer among women, with an age-adjusted incidence rate of 11.5 per lakh in men and 8.9 per lakh in women [2]. The high prevalence in this region is attributed to the widespread use of tobacco products, betel quid chewing, and alcohol consumption, combined with limited access to early diagnostic facilities and treatment centers [2,3].

The progression and prognosis of OSCC are influenced by complex interactions within the tumor microenvironment (TME), which comprises various cellular and non-cellular components [4]. Among these, cancer-associated fibroblasts (CAFs) have emerged as crucial modulators of tumor behavior [5]. CAFs develop from the transformation of normal fibroblasts into an activated phenotype, demonstrating remarkable plasticity and heterogeneity in their functions [6]. They are involved in various aspects of tumor progression, namely, extracellular matrix (ECM) remodelling, angiogenesis, and immune response modulation [7,8]. The identification and characterization of CAFs rely on the expression of specific markers, with alpha-smooth muscle actin (α-SMA) being one of the most reliable and widely used markers [9,10]. Studies show that the presence and distribution pattern of CAFs correlate with various clinicopathological parameters in OSCC [4,11]. But how its presence influences lymph node metastasis (LNM) and pathological tumor, node, and metastasis (pTNM) stage is still unclear [12].

LNM serves as one of the most considerable prognostic indicators in OSCC, with its presence substantially reducing the overall survival (OS) from ~80% to 40% [13]. Regional studies from Karnataka have reported nodal metastasis in 40-60% of OSCC cases at presentation, which is significantly higher than the global average of 30-35% [14].

The conventional tumor, node, and metastasis (TNM) staging does not fully portray the biological complexity of tumor progression, notably the role of stromal elements [3,15]. Recent studies suggest the incorporation of stromal markers, including CAF assessment, as an additional prognostic data [16].

The interaction between the CAF in stroma and tumor cells creates a permissive environment (TME), enabling tumor invasion and metastasis [11,17]. CAFs secrete various growth factors, cytokines, and matrix metalloproteinases (MMPs) that contribute to the formation of a pre-metastatic niche and enhance tumor invasiveness [18]. CAFs have also been implicated in the modulation of immune responses within the TME, influencing treatment and prognosis [19].

Various studies have demonstrated the association between CAF density and clinicopathological parameters in OSCC [20,21]. However, these have yielded conflicting results, possibly due to variations in their methodology, scoring system, and patient population [9]. Moreover, these studies have focused on isolated aspects of CAF biology, rather than a multivariate analysis [12,22].

Need for study

While the presence of CAF in stroma is a possible prognostic marker, the correlation between their distribution pattern and specific clinical consequences remains unexplored [6,7]. The existing literature principally focuses on CAF density as a separate parameter, without sufficient consideration of its association with other significant prognostic factors such as depth of invasion (DOI), pattern of invasion, and extracapsular extension (ECE) [14,23]. Therefore, this study aims to tackle this gap by examining the association between CAF density and various clinicopathological parameters simultaneously, providing a comprehensive understanding of their prognostic significance.

While several scoring systems for CAF assessment have been proposed, there is no consensus on the most clinically relevant method for evaluating CAF distribution and its prognostic implications [9,12]. Hence, this study employed a standardized scoring system based on the immunohistochemical expression of α-SMA, contributing to the establishment of more reliable criteria for CAF assessment.

Additionally, most existing studies have been conducted in different geographical and ethnic populations, with limited data available from the South Indian context, particularly Karnataka [2]. Given the high prevalence of OSCC in this region, with an annual incidence of approximately 8,000 new cases in Karnataka alone, and potential variations in tumor biology across different populations, this study provides valuable insights specific to the local patient population, optimizing treatment strategies and thereby improving patient outcomes.

Objectives

This study aimed to detect CAF using α-SMA in OSCC and to determine the association of CAF with LNM and pTNM staging in OSCC.

The oral cavity

During the fourth week of embryonic development, the pharyngeal or branchial arches develop, contributing to the development of the face, neck, and related structures. Each arch has an outer ectodermal layer, a mesodermal core, and an inner endodermal coating [24,25].

The oral cavity encompasses numerous anatomic sites and subsites vital for understanding OSCC. These comprise the buccal mucosa, floor of the mouth, hard palate, upper and lower gums, oral tongue, and retromolar trigone. Each site plays a different part in oral health and disease. The mucosa lines the inner cheeks, while the floor of the mouth supports the tongue. The hard palate forms the ridge of the mouth, and the oral tongue is the mobile part of the tongue, crucial for taste and speech. The retromolar trigone is the area behind the last molar, often a site for neoplasms. Each site presents unique challenges and prognoses. Hence, their understanding becomes vital for diagnosing and treating oral cancers [26,27].

## Materials and methods

Study design and setting

This was a retrospective analytical cross-sectional study conducted in the Department of Pathology at Sri Devaraj Urs Medical College, Kolar, Karnataka, India, from September 2022 to December 2023. The specimens were obtained from patients who had undergone surgical resection at the attached R.L. Jalappa Hospital and Research Centre. 

Sample size calculation

The sample size was calculated using Cochran's formula $\text{n}=\text{Z&sup2;}&times;\text{p}&times;\left(1-\text{p}\right)/\text{d&sup2;}$, where Z is the standard normal variate at 5% type 1 error (1.96), p is the expected proportion based on previous studies, and d is the absolute error or precision. 

The sample size was estimated based on 88.2% prevalence for α-SMA in OSCC as reported in Kellermann et al.'s study [7]. Considering an absolute error of 7% at a 95% confidence interval, the estimated sample size for the study was 88 cases of OSCC [20]. 

Fujii et al. reported CAF prevalence in 60.5% of OSCC cases in their study population of 60 patients [4].

Inclusion criteria

The study included histopathologically confirmed cases of OSCC treated by composite resection with modified radical neck dissection. Only primary OSCC cases with available paraffin-embedded tissue blocks and corresponding clinical and pathological data were included in the study.

Exclusion criteria

All the recurrent cases of oral carcinoma, those patients who received neoadjuvant chemotherapy for head and neck malignancy prior to surgical intervention, and second primary cancer cases were excluded.

Data collection procedure

Paraffin blocks and slides of histopathologically diagnosed OSCC cases were retrieved from the archives of the Department of Pathology. Patient demographic data, including age, gender, and anatomical location of the tumor, were collected from clinical records. All H&E-stained slides were reviewed by two pathologists independently to confirm the diagnosis and histopathological grade according to the Eighth Edition of the American Joint Committee on Cancer (AJCC) Staging of Head and Neck Cancer.

Immunohistochemical staining using mouse monoclonal (1A4) α-SMA antibody was performed on 4 μm sections of 10% neutral buffered formalin-fixed paraffin-embedded tissue samples. After deparaffinization in xylene and rehydration through a descending ethanol series at room temperature, antigen retrieval was performed using the heat retrieval method (microwave technique). Endogenous peroxidase activity was blocked using 3% hydrogen peroxide for five minutes. Sections were then incubated with the primary monoclonal antibody against α-SMA for 60 minutes at room temperature. The sections were then rinsed with Tris-buffered saline (TBS) three times, followed by incubation with horseradish peroxidase (HRP)-conjugated secondary antibody for 15-30 minutes at room temperature. Diaminobenzidine (DAB) was used as the chromogen for five minutes, and slides were counterstained by incubation with hematoxylin for five minutes. Ductal carcinoma of the breast tissue served as the positive control, while endothelial cells of blood vessels served as the internal positive control.

The α-SMA-stained (cytoplasmic stain) CAFs in the OSCC islands were counted using Kellermann et al.'s scoring system: Score 1 (−) (negative): when no staining or if <1% of CAFs were stained with α-SMA; Score 2 (+) (scanty): when >1% and <50% of CAFs were stained with α-SMA; and Score 3 (++) (abundant): when >50% of CAFs were stained with α-SMA.

Considering the distribution pattern of CAF, the arrangement of positive-stained cells was classified into three groups: (1) focal, which means focal CAF with no special arrangement in different areas of the tumor stroma; (2) network, which means interwoven network arrangement of CAF in the tumor stroma; and (3) spindle, which means spindled arrangement of CAF in one to three rows in the periphery of the neoplastic islands or the connective tissues [20].

Additionally, tumor-stroma ratio (TSR), worst pattern of invasion (WPOI), tumor budding (TB), and tumor-infiltrating lymphocytes (TILs) were assessed in each specimen.

Data analysis

The gathered data were imported into Microsoft Excel (Microsoft Corporation, Redmond, Washington, United States) and then examined using IBM SPSS Statistics for Windows, Version 23.0 (Released 2015; IBM Corp., Armonk, New York, United States). Descriptive statistics were employed to characterize the study population, including measures of central tendency (mean, median) and dispersion (standard deviation) for continuous variables such as age and DOI. Frequency distributions and percentages were calculated for categorical variables, including gender, tumor lateralization, histological differentiation, anatomical site, and various histopathological parameters. For analyzing associations between categorical variables, Pearson's chi-squared (χ²) test was utilized to assess statistical significance, with p-values <0.05 considered statistically significant. This approach was specifically applied to evaluate relationships between CAF scores and LNM, CAF architectural patterns and lymph node status, and CAF scores in relation to pTNM staging.

## Results

Eighty-eight cases of OSCC were studied, and the results are as follows: The quantitative analysis of CAFs utilizing α-SMA immunohistochemistry demonstrated a notable predominance of high CAF expression within the TME. Score 3, indicating abundant CAF (>50% positively stained cells), was observed in 49 (55.7%) cases, representing the majority of the specimens. Score 2, denoting scant CAF (1-50% positively stained cells), was identified in 36 (40.9%) cases. Conversely, Score 1, signifying negative or minimal CAF presence (<1% positively stained cells), was observed in only three (3.4%) cases. This suggested a significant tendency toward elevated CAF presence in the tumor stroma of OSCC, which may have profound implications for understanding the TME and its correlation with pathological staging and nodal metastasis (Table 1).

**Table 1:** Distribution of CAF score in OSCC CAF: cancer-associated fibroblast; OSCC: oral squamous cell carcinoma

CAF score	Frequency	Percent
1	3	3.4
2	36	40.9
3	49	55.7
Total	88	100

The morphological characterization of CAF within the TME revealed a distinctive architectural arrangement with potential implications for tumor-stromal interactions. The network pattern demonstrated predominance with 34 (38.6%) cases. Both focal and spindle configurations exhibited identical prevalence with 27 (30.7%) cases each (Table 2). 

**Table 2:** Distribution pattern of CAF in OSCC CAF: cancer-associated fibroblast; OSCC: oral squamous cell carcinoma

CAF distribution pattern	Frequency	Percent
Focal	27	30.7
Network	34	38.6
Spindle	27	30.7
Total	88	100

A substantial majority of specimens, specifically 59 (67%), exhibited a TSR <50%, indicating stromal predominance. Conversely, 29 (33%) cases presented with a TSR equal to or exceeding 50%, signifying tumor cell predominance (Table 3). 

**Table 3:** TSR in OSCC TSR: tumor-stroma ratio; OSCC: oral squamous cell carcinoma

TSR	Frequency	Percent
<50	59	67
≥50	29	33
Total	88	100

Table 4 enumerates the histomorphological assessment of the invasive frond characteristics, quantified through the WPOI types. Type 4 constituted the most prevalent category with 36 (40.9%) cases. Type 5, representing the most aggressive phenotype with marked and widespread cellular dissociation, accounted for 26 (29.5%) cases. Type 3 was observed in 20 (22.7%) cases, and Type 2 demonstrated minimal representation with merely six (6.8%) cases. 

**Table 4:** Distribution of WPOI types in OSCC WPOI: worst pattern of invasion; OSCC: oral squamous cell carcinoma

WPOI types	Frequency	Percent
1	-	-
2	6	6.8
3	20	22.7
4	36	40.9
5	26	29.5
Total	88	100

The quantitative evaluation of TB (Table 5) revealed a stratified distribution across the established categorical thresholds. The predominant category, comprising specimens with <5 buds/high-power field (hpf), accounted for 40 (45.5%) cases. Intermediate burden, characterized by 5-10 buds/hpf, was observed in 35 (39.8%) specimens. High-intensity TB with >10 buds/hpf was seen in 13 (14.8%) cases. 

**Table 5:** Quantitative analysis of TB in OSCC TB: tumor budding; OSCC: oral squamous cell carcinoma

TB	Frequency	Percent
<5	40	45.5
5-10	35	39.8
>10	13	14.7
Total	88	100

The immunological microenvironment assessment, quantified as TILs (Table 6), demonstrated a predominant intermediate immunological response. The majority of specimens, i.e., 51 (58%), exhibited a moderate TIL density ranging from 20 to 80 lymphocytes/hpf. A substantial minority, 33 (37.5%) cases, demonstrated limited lymphocytic infiltration, <20 lymphocytes/hpf, potentially indicating an immune evasion or suppression mechanism. Notably, robust lymphocytic infiltration, >80 lymphocytes/hpf, represented an exceptional finding, observed in merely four (4.5%) specimens.

**Table 6:** Quantitative distribution of TILs in OSCC TILs: tumor-infiltrating lymphocytes; OSCC: oral squamous cell carcinoma

TILs (%)	Frequency	Percent
<20	33	37.5
20-80	51	58
>80	4	4.5
Total	88	100

The analysis of CAF distribution pattern in relation to pTNM staging (Table 7) demonstrated a statistically significant association (χ²=26.716; p=0.001). In Stage I disease, CAFs exhibited both focal and spindle patterns, each with two (50%) cases, with no cases displaying a network pattern. Stage II predominantly featured focal CAF distribution in 10 (55.6%) cases, followed by network pattern in six (33.3%) cases, while spindle arrangement was observed in two (11.1%) cases. In Stage III disease, focal pattern remained predominant in 12 (42.9%) cases, network pattern in 10 (35.7%) cases, and spindle arrangement in six (21.4%) cases. Conversely, Stage IVA revealed a notable shift toward network pattern, observed in 16 (55.2%) cases, spindle arrangement in 10 (34.5%) cases, and focal in three (10.3%) cases. Stage IVB demonstrated the most striking pattern shift, with spindle arrangement occurring in seven (77.8%) cases, network pattern in two (22.2%) cases, and complete absence of focal pattern of arrangement. This progressive transition from focal to network and ultimately spindle CAF pattern of arrangement, with advancing TNM stages, suggests a correlation between CAF distribution pattern and tumor progression. 

**Table 7:** Association between CAF distribution pattern and pTNM stage Chi-squared value: 26.716; p-value: 0.001 CAF: cancer-associated fibroblast; pTNM: pathological tumor, node, and metastasis

pTNM stage	CAF distribution pattern		
	Focal (n=27)	Network (n=34)	Spindle (n=27)
I	2 (50%)	0 (0%)	2 (50%)
II	10 (55.6%)	6 (33.3%)	2 (11.1%)
III	12 (42.9%)	10 (35.7%)	6 (21.4%)
IVA	3 (10.3%)	16 (55.2%)	10 (34.5%)
IVB	0 (0%)	2 (22.2%)	7 (77.8%)

The relationship between TSR and CAF score (Table 8) is statistically significant (χ²=28.077; p<0.0001), suggesting a non-stochastic interrelationship between stromal proportion and fibroblastic activation. All the stroma-rich cases (TSR <50) showed significant CAF presence, with CAF Score 2 in 15 (25.4%) cases and Score 3 in 44 (74.6%) cases. At the same time, stroma-poor cases (TSR ≥50) exhibited CAF Score 1 in three (10.3%) cases, CAF Score 2 in 21 (72.4%) cases, and CAF Score 3 in five (17.2%) cases. These findings suggest a potential relationship between the stromal volume and CAF activity, where abundant stroma heightened CAF activation, indicating both quantitative and qualitative tumor-stromal interactions. The preponderance of higher CAF scores in stroma-dominant cases substantiates an enhanced myofibroblastic differentiation, augmenting the invasive and metastatic capabilities of OSCC through concentrated paracrine signalling pathways and ECM remodelling activities.

**Table 8:** Association between TSR and CAF score in OSCC Chi-squared value: 28.077; p-value: <0.0001 TSR: tumor-stroma ratio; CAF: cancer-associated fibroblast; OSCC: oral squamous cell carcinoma; n: number

TSR	CAF score		
	1 (n=3)	2 (n=36)	3 (n=49)
<50	0 (0%)	15 (25.4%)	44 (74.6%)
≥50	3 (10.3%)	21 (72.4%)	5 (17.2%)

The relationship between WPOI and TB (Table 9) demonstrates a robust statistical correlation (χ²=48.937; p<0.0001). Among the WPOI Type 2, all six (100%) cases demonstrated minimal TB (<5 buds/hpf). Similarly, in WPOI Type 3, 19 (95%) cases exhibited minimal TB, and only one (5%) case displayed moderate budding (5-10 buds/hpf). With WPOI Type 4, 15 (41.7%) cases each demonstrated minimal and moderate budding, and six (16.7%) cases exhibited extensive budding (>10 buds/hpf). The most aggressive invasive pattern, WPOI Type 5, displayed a stark contrast to the rest, with a predominance of moderate budding in 19 (73.1%) cases, extensive budding (>10) in seven (26.9%) cases, and complete absence of minimal budding. This progressive increase in TB frequency and advancing WPOI types suggests a synchronous evolution of invasive patterns and TB phenomena, reflecting a coordinated molecular mechanism culminating in OSCC progression.

**Table 9:** Association between WPOI and TB Chi-squared value: 48.937; p-value: <0.0001 WPOI: worst pattern of invasion; TB: tumor budding; n: number

WPOI	TB		
	<5 (n=40)	>10 (n=13)	5-10 (n=35)
2	6 (100%)	0 (0%)	0 (0%)
3	19 (95%)	0 (0%)	1 (5%)
4	15 (41.7%)	6 (16.7%)	15 (41.7%)
5	0 (0%)	7 (26.9%)	19 (73.1%)

The non-random statistically significant relationship (χ²=11.555; p=0.021) between TB intensity and CAF score is presented in Table 10. In cases with minimal TB (<5), moderate CAF activity was observed, with CAF Score 2 identified in 23 (57.5%) cases, CAF Score 3 in 16 (40%) cases, and CAF Score 1 in one (2.5%) case. Cases with moderate TB displayed a markedly different distribution pattern, with the highest CAF activity observed in most, i.e., 26 (74.3%) cases, CAF Score 2 in seven (20%) cases, and CAF Score 1 in only two (5.7%) cases. In specimens manifesting extensive TB (>10), CAF Scores 2 and 3 were identified in six (46.2%) and seven (53.8%) cases, respectively. This progressive increase in CAF activation with escalating TB frequency suggests a coordinated epithelial-mesenchymal interaction facilitating invasive tumor behavior. This synergistic stromal activation and epithelial disaggregation at the invasive front constitutes an aggressive OSCC phenotype.

**Table 10:** Association between TB and CAF score Chi-squared value: 11.555; p-value: 0.021 TB: tumor budding; CAF: cancer-associated fibroblast; n: number

TB	CAF score		
	1 (n=3)	2 (n=36)	3 (n=49)
<5	1 (2.5%)	23 (57.5%)	16 (40%)
5-10	2 (5.7%)	7 (20%)	26 (74.3%)
>10	0 (0%)	6 (46.2%)	7 (53.8%)

The analysis examining the association between TB intensity and LNM status (Table 11) reveals a statistically significant relationship (χ²=6.107; p=0.047), suggesting that TB may serve as a histopathological predictor of nodal involvement in OSCC. In specimens exhibiting minimal TB (<5), an equal distribution between metastatic and non-metastatic cases was observed, with 20 (50%) specimens in each category. Among specimens characterized by moderate TB (5-10), a slight predominance of non-metastatic cases was noted, with 19 (54.3%) specimens demonstrating absence of nodal metastasis and 16 (45.7%) exhibiting nodal involvement. The most striking differential pattern emerged in specimens with extensive TB (>10), wherein a substantial predominance of metastatic cases was observed, with 11 (84.6%) specimens demonstrating lymph node involvement, compared to merely two (15.4%) cases without nodal metastasis. This progressive increase in the proportion of metastatic cases with escalating TB intensity underscores the potential utility of TB as a histopathological biomarker for nodal metastasis risk stratification. The marked predilection for lymph node involvement in specimens with extensive budding suggests that the disaggregation of epithelial cells at the invasive tumor front may facilitate lymphovascular invasion (LVI), thereby promoting metastatic dissemination via lymphatic channels. This observation corroborates previous investigations implicating TB as a manifestation of partial epithelial-mesenchymal transition (EMT) conducive to enhanced cellular motility and metastatic propensity. 

**Table 11:** Relationship between TB and LNM Chi-squared value: 6.107; p-value: 0.047 TB: tumor budding; LNM: lymph node metastasis; n: number

TB	LNM	
	Yes (n=47)	No (n=41)
<5	20 (50%)	20 (50%)
5-10	16 (45.7%)	19 (54.3%)
>10	11 (84.6%)	2 (15.4%)

**Figure 1:**
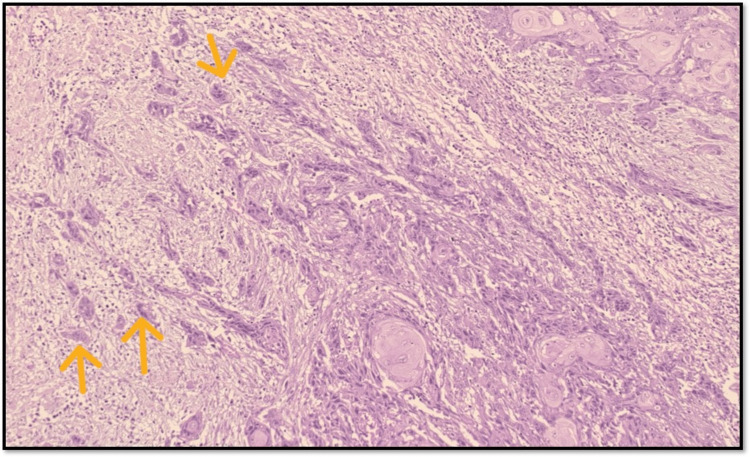
Microphotograph showing tumor buds (original magnification, 100×)

TB is characterized by the presence of isolated single cells or small clusters of cells at the invasive front of tumors and has garnered attention as a significant prognostic factor in OSCC. Its presence is associated with aggressive tumor behavior and poorer clinical outcomes, making it a crucial consideration in the prognostic evaluation of OSCC (Figure 1).

**Figure 2:**
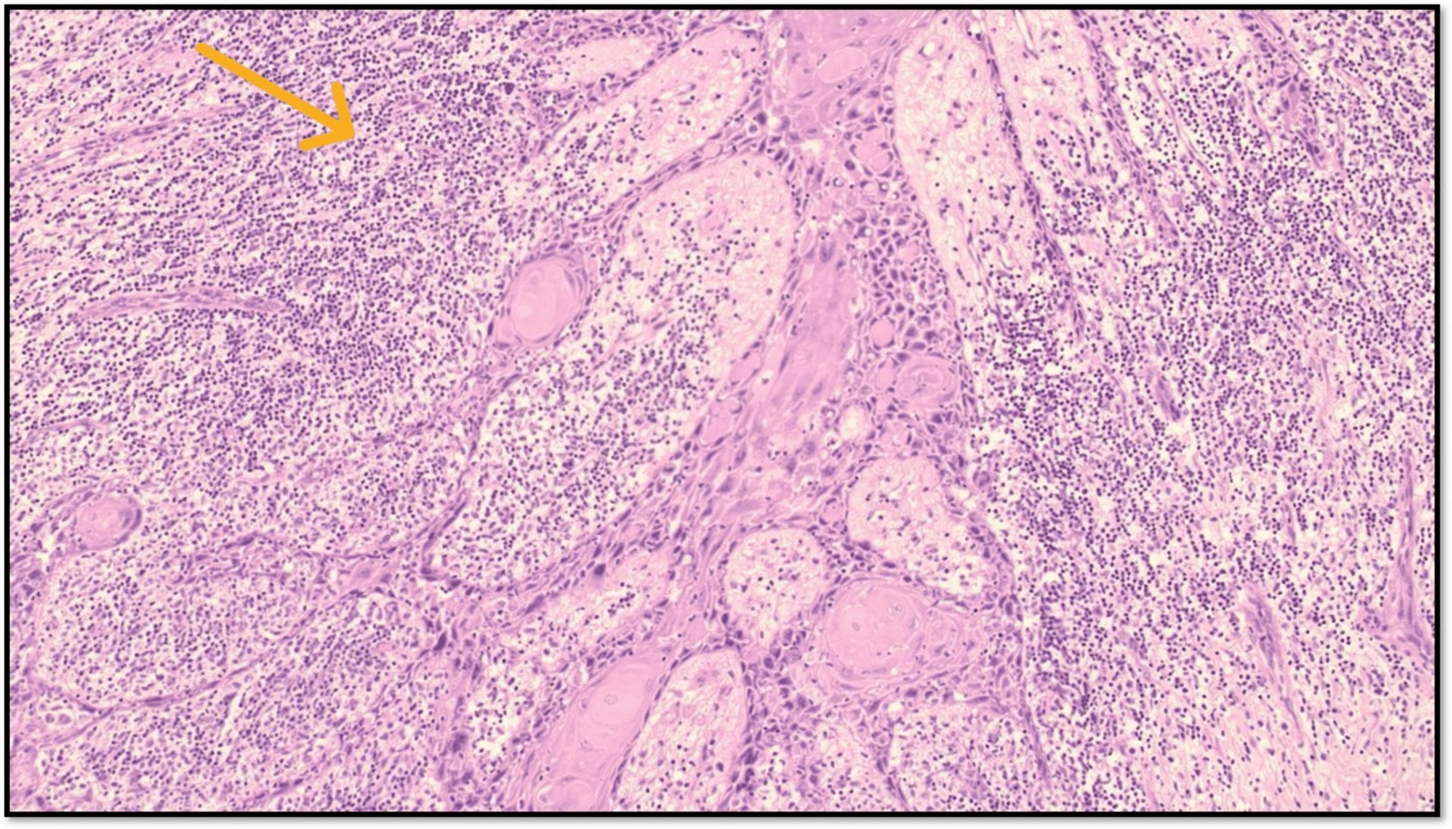
Microphotograph showing intratumoral moderate TILs (original magnification, 100×) TILs: tumor-infiltrating lymphocytes

TILs have emerged as significant prognostic factors in OSCC, reflecting the body's immune response to tumor cells. Their presence indicates how the immune system interacts with cancer, influencing both the progression and the potential response to therapies (Figure 2). 

**Figure 3:**
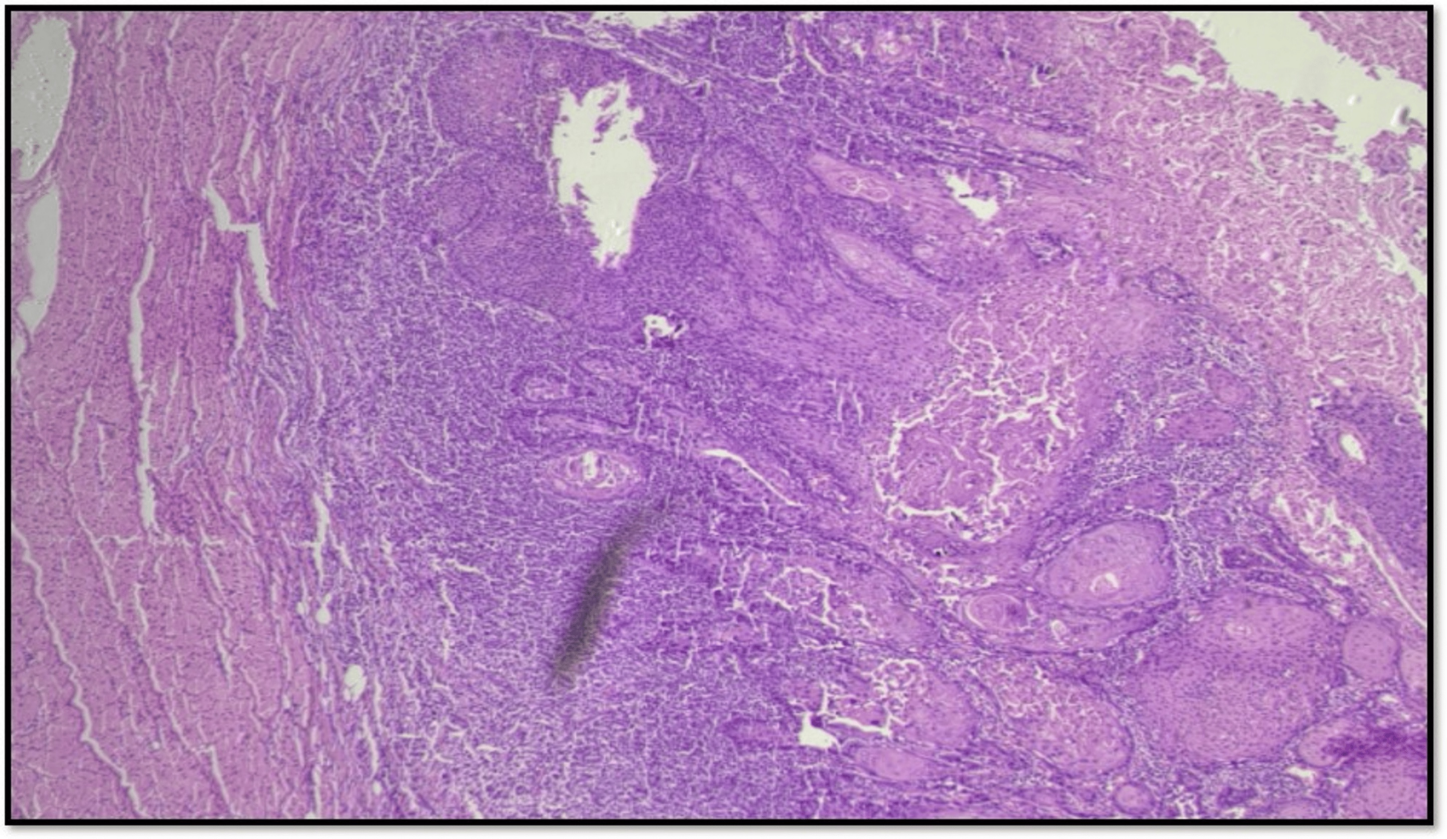
Microphotograph showing WPOI Type 3 (original magnification, scanner view) WPOI: worst pattern of invasion

Type 3 WPOI is defined as large tumor islands, made up of at least 15 cells (Figure 3).

**Figure 4:**
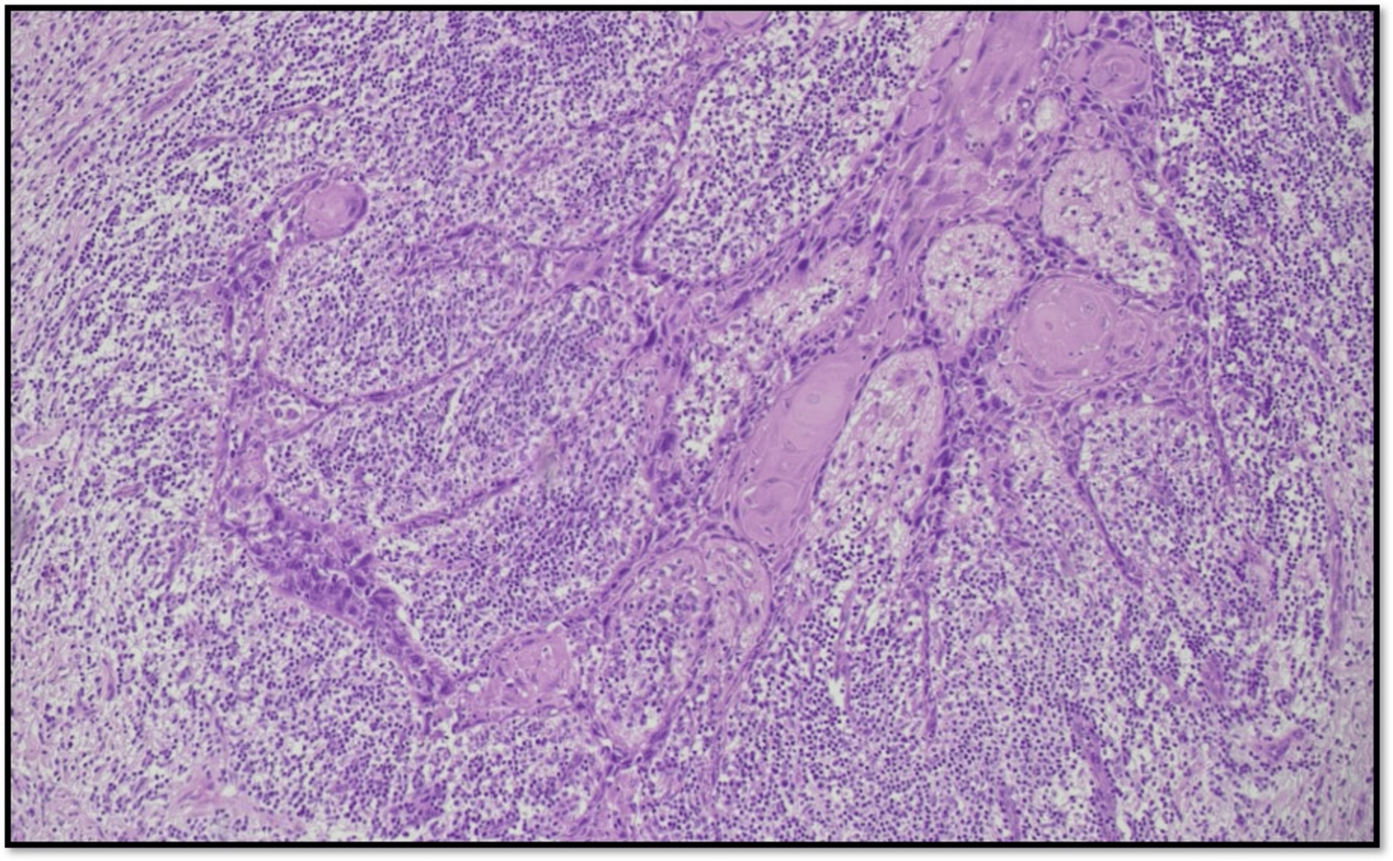
Microphotograph showing WPOI Type 4 (original magnification, scanner view) WPOI: worst pattern of invasion

Type 4 WPOI is defined as a small tumor island, consisting of <15 cells (Figure 4). 

**Figure 5:**
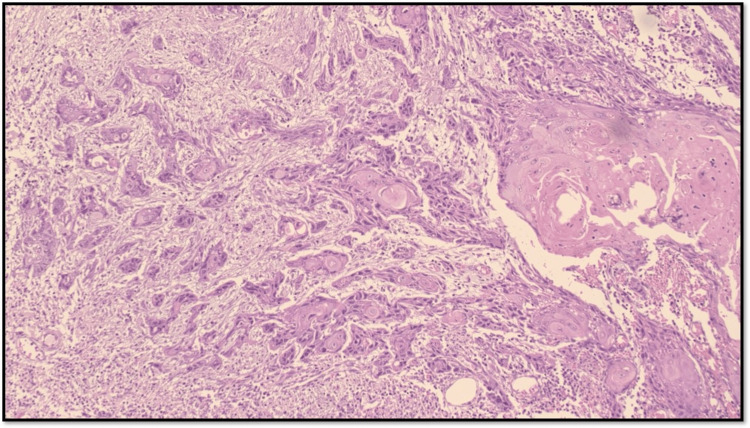
Microphotograph showing WPOI Type 5 (original magnification, 100×) WPOI: worst pattern of invasion

Type 5 WPOI is defined as the presence of tumor satellites that are 1 mm away from the main tumor (Figure 5). 

The important prognostic markers in OSCC, perineural invasion (PNI), and LVI greatly affect cancer patient outcomes, including survival as well as recurrence. These invading patterns influence treatment plans besides revealing the aggressiveness of the cancer.

**Figure 6:**
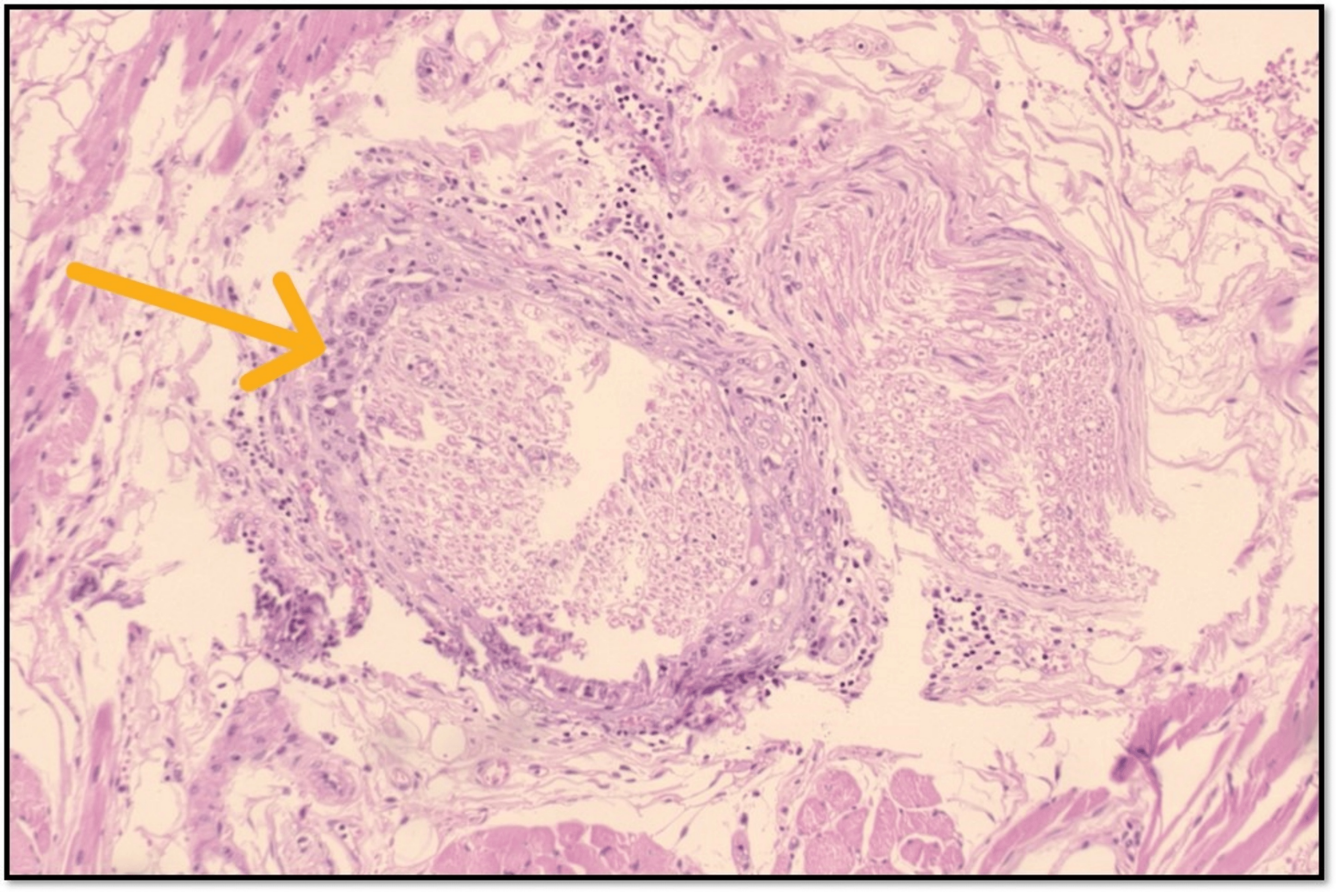
Microphotograph showing evidence of PNI (original magnification, 200×) PNI: perineural invasion

PNI (Figure 6) results from cancer cells invading the area around neurons and thereby enabling the tumor cells to migrate via nerve pathways, thereby acting as an independent predictive factor for local and regional failure.

**Figure 7:**
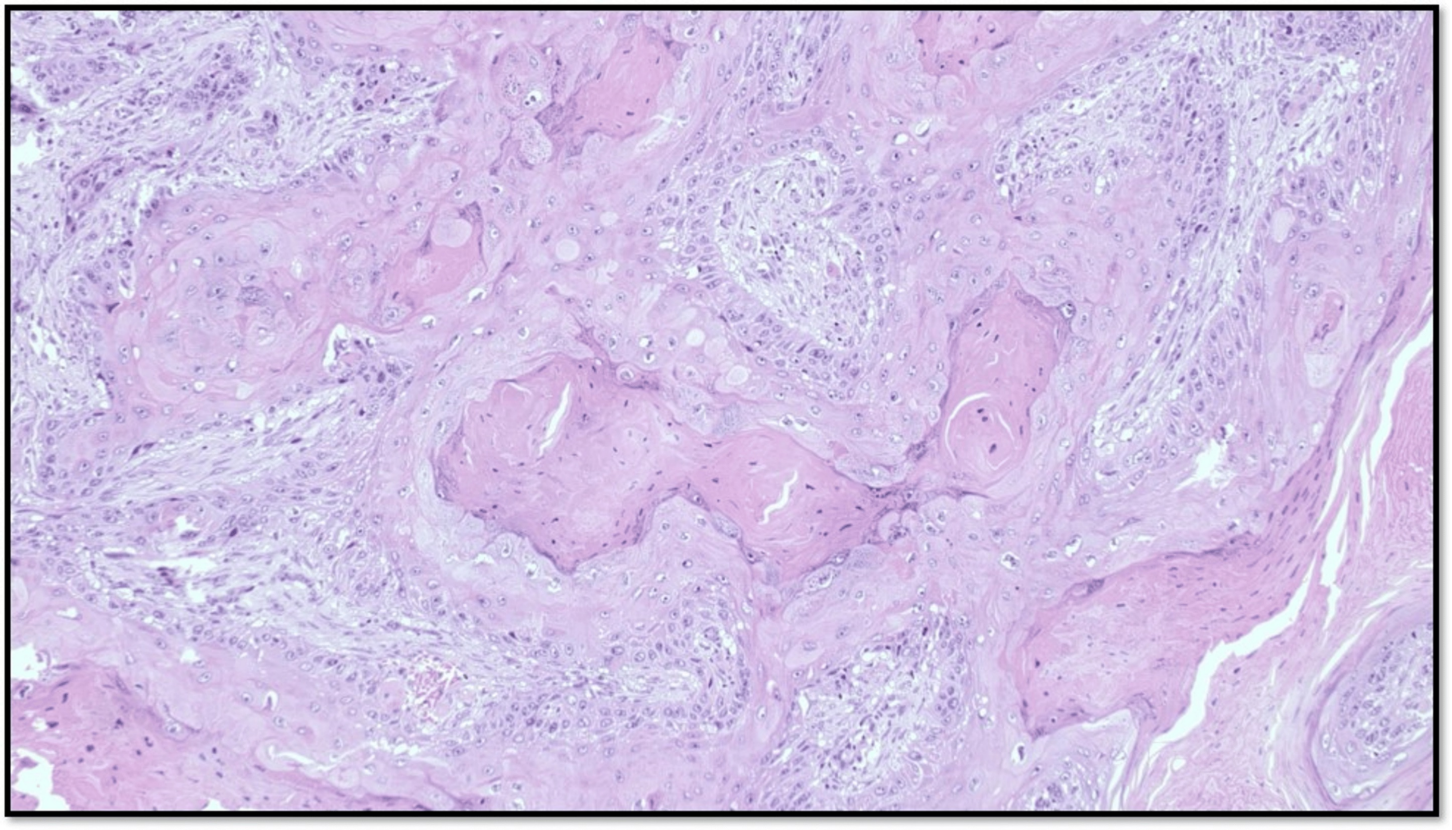
Microphotograph showing tumor involvement in bone (original magnification, 200×)

A major prognostic determinant among all, bone metastasis in OSCC, points to a more aggressive cancer progression and worse patient outcomes among the patients. Advanced tumors as well as the invasion of cancer cells into the bone are linked and may also complicate therapy as well as lower survival chances (Figure 7). 

CAFs contribute to the EMT, a process by which epithelial cells acquire mesenchymal, invasive characteristics. This transition is facilitated by CAF-derived factors like transforming growth factor-beta (TGF-β), which activate signalling pathways involved in EMT and enhance the metastatic potential of OSCC cells [22].

CAFs are integral to OSCC pathogenesis, influencing tumor progression through ECM remodelling, immune modulation, and angiogenesis. Their complex interactions with cancer cells and the microenvironment make them attractive targets for therapeutic intervention, with ongoing research aimed at unravelling the molecular pathways involved in their diverse functions.

**Figure 8:**
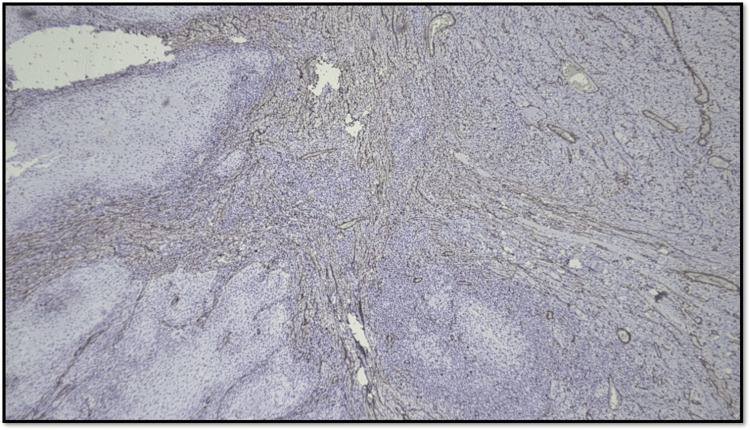
Microphotograph showing CAF Score 2 (original magnification, scanner view) CAF: cancer-associated fibroblast

Figure 8 shows an OSCC case with 1-50% CAF stained with α-SMA, which is Score 2.

**Figure 9:**
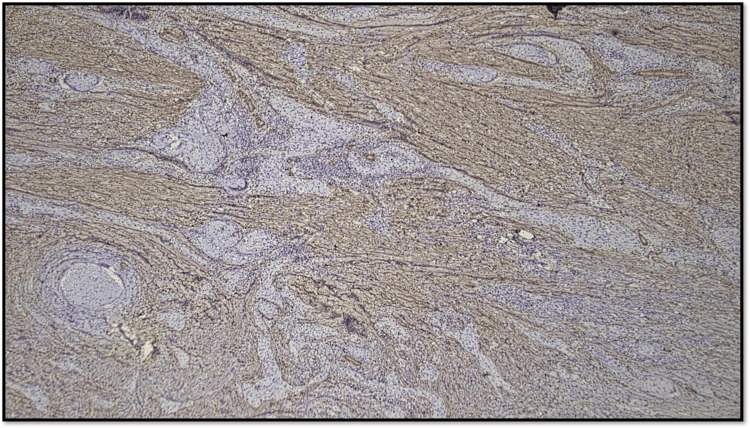
Microphotograph showing CAF Score 3 (original magnification, 200×) CAF: cancer-associated fibroblast

Figure 9 shows abundant CAF (>50% CAF stained with α-SMA) in the tumor stroma.

**Figure 10:**
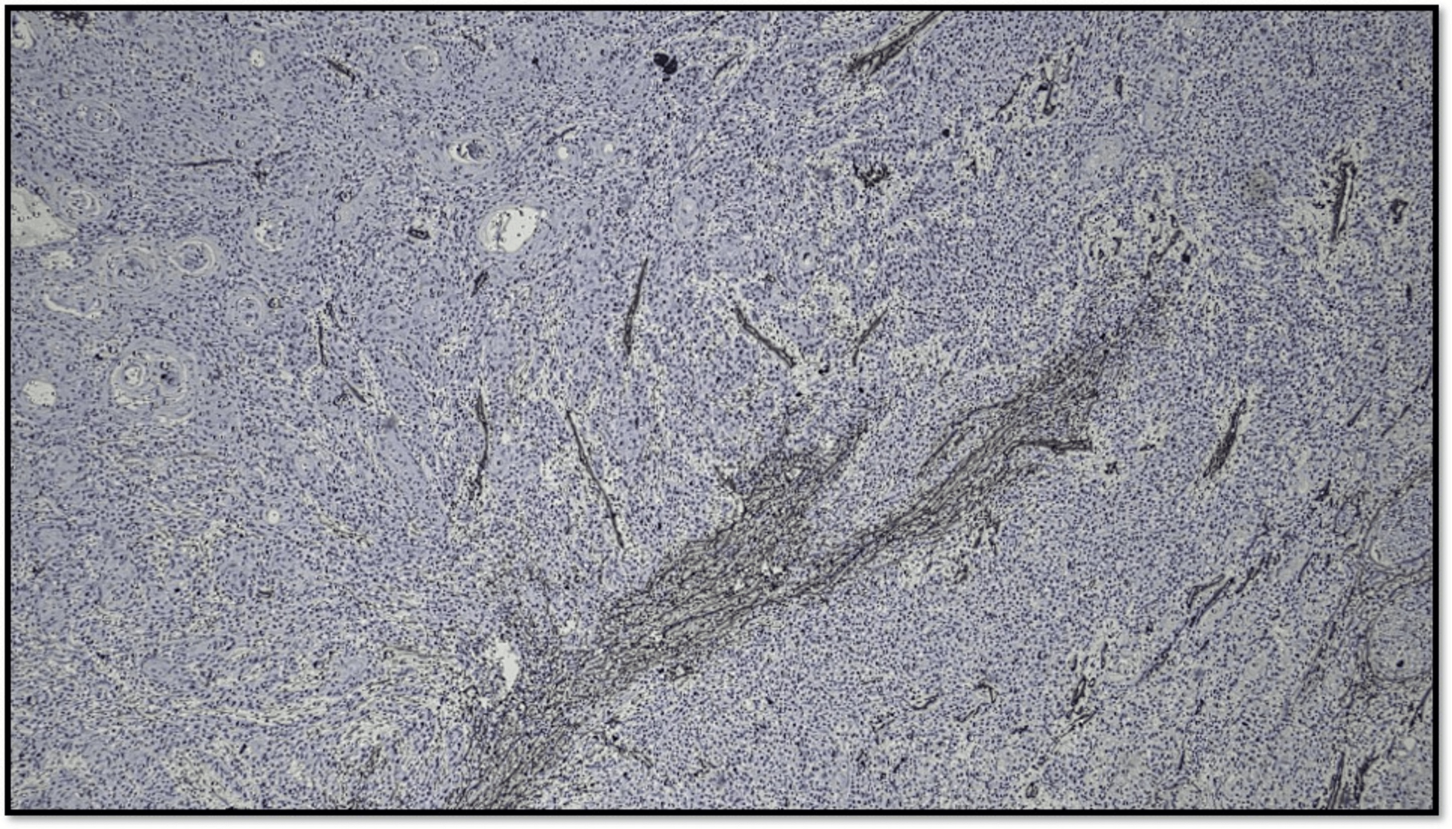
Microphotograph showing focal pattern of distribution of CAF (original magnification, 100×) CAF: cancer-associated fibroblast

**Figure 11:**
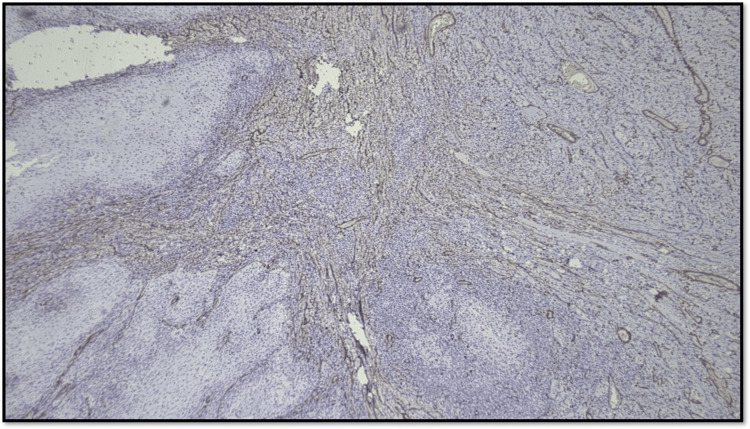
Microphotograph showing network pattern of distribution of CAF (original magnification, 100×) CAF: cancer-associated fibroblast

**Figure 12:**
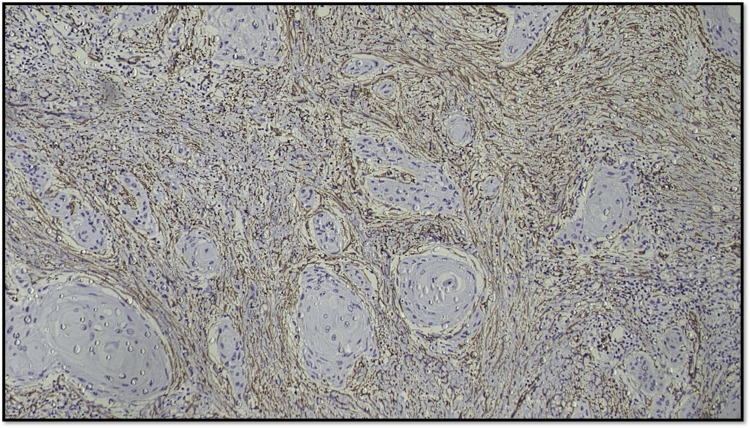
Microphotograph showing spindle pattern of distribution of CAF (original magnification, 200×) CAF: cancer-associated fibroblast

Considering the distribution pattern of CAF, the arrangement of positive-stained cells was classified into three groups (Figures 10-12): (1) focal, which means focal CAF with no special arrangement in different areas of the tumor stroma; (2) network, which means interwoven network arrangement of CAF in the tumor stroma; and (3) spindle, which means spindled arrangement of CAF in one to three rows in the periphery of the neoplastic islands or the connective tissues [20].

## Discussion

The gender disparity in our cohort reflects region-specific risk factor exposure, relating to cultural practices of tobacco use among women. Borse et al. noted similar gender variations in certain regions of India where tobacco chewing is common among women [2].

The anatomical distribution of OSCC in our cohort, with buccal mucosa representing 50% of cases, reflects regional variations in OSCC predilection sites, potentially influenced by specific risk factors prevalent in the study population. This differs from Western populations, where the tongue and floor of the mouth are typically more common sites. Fatima et al. analyzed 121 OSCC cases from Pakistan and similarly found the buccal mucosa as the predominant site (56.2%), attributing this to prevalent smokeless tobacco habits in the region [15].

Among the node metastatic specimens, spindle configuration predominated (38.3%), whereas non-metastatic cases showed preference for network (41.5%) and focal (36.6%) patterns. This suggests that the spatial organization and morphological characteristics of CAFs have greater prognostic relevance than their absolute quantity. Luksic et al. found that the arrangement pattern of myofibroblasts in 152 OSCC patients in Croatia was more predictive of occult regional metastasis than their density alone (p<0.001), with spindle-shaped myofibroblasts at the invasive front correlating with higher metastatic potential [21].

Our analysis of the CAF distribution patterns in relation to pTNM staging demonstrated a strong statistically significant association (p=0.001). A distinct progression was observed from predominantly focal patterns in early stages to network patterns in Stage IVA and spindle arrangements in Stage IVB. This finding suggests an evolutionary shift in CAF architectural organization with disease progression, potentially reflecting dynamic alterations in tumor-stromal interactions during cancer advancement. Zhang et al. similarly documented altered CAF morphology and distribution with increasing tumor stage in 124 OSCC specimens from Chinese patients, with distinct arrangements correlating with invasive potential and metastatic behavior (p<0.01) [28].

We also found that specimens with low TSR (stroma-rich) exhibited higher CAF score (CAF Score 3 in 74.6%), while specimens with high TSR (stroma-poor) showed lower CAF score (CAF Score 2 in 72.4%) (p<0.0001). This suggests that abundant stroma enhances CAF functionality and activity, trending towards aggressive behavior of the tumor. Takabatake et al. reported similar observations in 60 OSCC specimens from Japan, noting that CAF concentration rather than absolute number correlated with invasive potential (p<0.05) [24].

Our study demonstrated a significant correlation between TB intensity and CAF score (p=0.021), with increasing TB associated with higher CAF activity. This finding suggests a synergistic relationship between CAFs and TB, potentially mediated through EMT mechanisms. Similar associations were reported by Ko et al., who found that CAF index significantly correlated with TB in 112 OSCC patients in Taiwan (p<0.001), proposing that CAFs facilitate collective cell migration at the invasive front [16].

Furthermore, TB demonstrated a significant association with nodal metastasis (p=0.047), with 84.6% of specimens exhibiting extensive budding (>10) and nodal involvement. This progressive increase in metastatic cases with escalating TB intensity underscores the potential utility of TB as a histopathological biomarker for nodal metastasis risk stratification. Comparable findings were reported by Ramasubramanian et al., who analyzed 122 OSCC cases from South India and found that higher TB significantly predicted nodal metastasis (p<0.001) [14].

The analysis of WPOI and TB revealed a strong correlation (p<0.0001), with more aggressive invasion patterns (WPOI Types 4 and 5) associated with higher TB. In specimens with WPOI Type 5, 73.1% exhibited moderate budding, and the remaining 26.9% showed extensive budding. This suggests that the invasion pattern and TB represent interrelated aspects of tumor progression, potentially driven by common molecular mechanisms. Khan et al. similarly found a significant association between the pattern of invasion and other histopathological parameters in 53 OSCC cases from India (p<0.05), supporting the concept that invasion patterns reflect fundamental biological properties of tumor aggressiveness [23].

Our findings regarding TILs revealed a predominant intermediate immunological response, with 58% of specimens exhibiting moderate TIL density. This observation, coupled with the significant CAF presence, suggests complex immunomodulatory interactions within the TME. Takahashi et al. demonstrated that CAFs promote an immunosuppressive microenvironment through the induction of pro-tumoral macrophages in 60 OSCC specimens from Japan (p<0.01), highlighting the role of CAFs in immune evasion [8]. Similarly, Sun et al. reviewed evidence that chronic inflammation influences OSCC progression through various immunosuppressive mechanisms, many of which involve CAF-mediated pathways [19].

Keeping in mind the right dexterity of the hand, which conveniently allows the placement of tobacco quid in the left lower quadrant of the mouth, accounts for the involvement of the left side of the buccal mucosa and lower alveolus.

The significant association between TB and LNM (p=0.047) has substantial implications for clinical decision-making, particularly in early-stage OSCC cases where occult nodal metastasis remains a critical challenge. Our finding that 84.6% of specimens with extensive TB (>10) exhibited nodal involvement suggests that incorporating TB assessment into routine histopathological evaluation could improve the accuracy of predicting lymph node status. This could guide more informed decisions regarding the necessity of elective neck dissection in clinically node-negative patients, potentially reducing both under-treatment and over-treatment. Particularly in resource-limited settings where advanced imaging modalities may be less accessible, TB assessment represents a cost-effective approach to risk stratification.

The strong association between WPOI and TB (p<0.0001) further reinforces the value of detailed histomorphological assessment of the invasive front. The complete absence of minimal budding in specimens with WPOI Type 5 highlights how these parameters could be evaluated in conjunction to better predict aggressive behavior. Implementing standardized reporting of both WPOI and TB could provide clinicians with more comprehensive risk assessment tools that better reflect the biological behavior of individual tumors, potentially allowing for more personalized treatment planning.

The relationship between TSR and CAF score (p<0.0001) supports the conventional understanding of stromal contribution to tumor progression and suggests both qualitative and quantitative assessment of CAF functionality. This finding has implications for developing targeted therapeutic strategies aimed at modulating CAF activity and depleting stromal components. Potential approaches could include inhibiting specific CAF-derived factors or disrupting CAF-tumor cell communication pathways, which might prove more effective than strategies targeting gross stromal reduction.

## Conclusions

The findings of this study offer several clinically significant insights that could potentially transform the management approaches for OSCC patients. The strong correlation between CAF architectural patterns and TNM staging (p=0.001) presents an opportunity for enhancing prognostic assessment beyond conventional parameters. The progression from focal patterns in early stages to network patterns in Stage IVA and spindle arrangements in Stage IVB suggests that CAF pattern evaluation could serve as a complementary histopathological marker for tumor aggressiveness. This approach could potentially identify patients at higher risk for disease progression who might benefit from more aggressive therapeutic strategies despite otherwise favorable conventional prognostic indicators.

Integrating CAF-related markers into prognostic models may improve the predictive value beyond traditional clinicopathological staging. Hence, by combining all our findings, it can be suggested that routine immunohistochemical staining for α-SMA to identify CAFs, coupled with detailed assessment of their distribution patterns, could significantly enhance the prognostic information derived from conventional histopathological examination. This approach could be particularly valuable in settings where advanced molecular testing is limited, offering an accessible method to improve risk stratification.

Given the accessibility and relatively low cost of immunohistochemistry compared to molecular techniques, the implementation of routine CAF assessment in OSCC specimens represents a feasible approach to enhancing prognostic information in various clinical settings. Standardization of CAF evaluation methodologies would be essential to ensure consistency and reproducibility, potentially leading to the development of consensus guidelines for CAF assessment in OSCC similar to those established for other histopathological parameters.

## References

[1] Bray F, Laversanne M, Sung H, Ferlay J, Siegel RL, Soerjomataram I, Jemal A (2024). Global cancer statistics 2022: GLOBOCAN estimates of incidence and mortality worldwide for 36 cancers in 185 countries. CA Cancer J Clin.

[2] Borse V, Konwar AN, Buragohain P (2020). Oral cancer diagnosis and perspectives in India. Sens Int.

[3] Majumdar B, Patil S, Sarode SC, Sarode GS, Rao RS (2017). Clinico-pathological prognosticators in oral squamous cell carcinoma: an update. Transl Res Oral Oncol.

[4] Fujii N, Shomori K, Shiomi T, Nakabayashi M, Takeda C, Ryoke K, Ito H (2012). Cancer-associated fibroblasts and CD163-positive macrophages in oral squamous cell carcinoma: their clinicopathological and prognostic significance. J Oral Pathol Med.

[5] Wright K, Ly T, Kriet M, Czirok A, Thomas SM (2023). Cancer-associated fibroblasts: master tumor microenvironment modifiers. Cancers (Basel).

[6] Arebro J, Lee CM, Bennewith KL, Garnis C (2024). Cancer-associated fibroblast heterogeneity in malignancy with focus on oral squamous cell carcinoma. Int J Mol Sci.

[7] Bienkowska KJ, Hanley CJ, Thomas GJ (2021). Cancer-associated fibroblasts in oral cancer: a current perspective on function and potential for therapeutic targeting. Front Oral Health.

[8] Takahashi H, Sakakura K, Kudo T, Toyoda M, Kaira K, Oyama T, Chikamatsu K (2017). Cancer-associated fibroblasts promote an immunosuppressive microenvironment through the induction and accumulation of protumoral macrophages. Oncotarget.

[9] Dourado RC, Porto LP, Leitão ÁCG, Cerqueira PS, Dos Santos JN, Ramalho LM, Xavier FC (2018). Immunohistochemical characterization of cancer-associated fibroblasts in oral squamous cell carcinoma. Appl Immunohistochem Mol Morphol.

[10] Pinisetti S, Tadi D, Manyam R, Alla R (2021). Immunohistochemical evaluation of myofibroblasts in oral epithelial dysplasia and oral squamous cell carcinoma. J Oral Maxillofac Pathol.

[11] Bello IO, Vered M, Dayan D (2011). Cancer-associated fibroblasts, a parameter of the tumor microenvironment, overcomes carcinoma-associated parameters in the prognosis of patients with mobile tongue cancer. Oral Oncol.

[12] Datar UV, Kale AD, Angadi PV, Hallikerimath S, Deepa M, Desai KM (2022). Role of cancer-associated fibroblasts in oral squamous cell carcinomas, surgical margins, and verrucous carcinomas: an immunohistochemical study. J Clin Transl Res.

[13] Bernasconi M, Bilic A, Kauke-Navarro M, Safi AF (2023). Nodal tumor volume as a prognostic factor for oral squamous cell carcinoma-a systematic review. Front Oral Health.

[14] Ramasubramanian S, Pandiar D, Krishnan RP, Ramalingam K, Bologna-Molina R (2023). Correlation of bony invasion with nodal metastasis, pattern of invasion and survival in oral squamous cell carcinoma: a retrospective analysis of 122 primary cases from oral cancer centre of South India. Cureus.

[15] Fatima J, Fatima E, Mehmood F, Ishtiaq I, Khan MA, Khurshid HM, Kashif M (2024). Comprehensive analysis of oral squamous cell carcinomas: clinical, epidemiological, and histopathological insights with a focus on prognostic factors and survival time. Cureus.

[16] Ko YC, Lai TY, Hsu SC (2020). Index of cancer-associated fibroblasts is superior to the epithelial-mesenchymal transition score in prognosis prediction. Cancers (Basel).

[17] Li H, Zhang J, Chen SW (2015). Cancer-associated fibroblasts provide a suitable microenvironment for tumor development and progression in oral tongue squamous cancer. J Transl Med.

[18] Shan Q, Takabatake K, Omori H (2021). Stromal cells in the tumor microenvironment promote the progression of oral squamous cell carcinoma. Int J Oncol.

[19] Sun Y, Liu N, Guan X, Wu H, Sun Z, Zeng H (2016). Immunosuppression induced by chronic inflammation and the progression to oral squamous cell carcinoma. Mediators Inflamm.

[20] Kellermann MG, Sobral LM, da Silva SD (2007). Myofibroblasts in the stroma of oral squamous cell carcinoma are associated with poor prognosis. Histopathology.

[21] Luksic I, Suton P, Manojlovic S, Virag M, Petrovecki M, Macan D (2015). Significance of myofibroblast appearance in squamous cell carcinoma of the oral cavity on the occurrence of occult regional metastases, distant metastases, and survival. Int J Oral Maxillofac Surg.

[22] Vered M, Dobriyan A, Dayan D (2010). Tumor-host histopathologic variables, stromal myofibroblasts and risk score, are significantly associated with recurrent disease in tongue cancer. Cancer Sci.

[23] Khan SJ, Gawande M, Hande AH, Patil SK, Sonone AM (2024). Correlation of pattern of invasion, stromal inflammation and lymphovascular invasion with histopathological grading in oral squamous cell carcinoma: a retrospective study. Cureus.

[24] Takabatake K, Kawai H, Omori H (2020). Impact of the stroma on the biological characteristics of the parenchyma in oral squamous cell carcinoma. Int J Mol Sci.

[25] Gupta A, Malhotra G, Akadiri O, Jackson IT (2010). Head and neck embryology and anatomy. Plastic and Reconstructive Surgery.

[26] Farhood Z, Simpson M, Ward GM, Walker RJ, Osazuwa-Peters N (2019). Does anatomic subsite influence oral cavity cancer mortality? A SEER database analysis. Laryngoscope.

[27] Kerker FA, Adler W, Brunner K (2018). Anatomical locations in the oral cavity where surgical resections of oral squamous cell carcinomas are associated with a close or positive margin-a retrospective study. Clin Oral Investig.

[28] Zhang JY, Zhu WW, Wang MY, Zhai RD, Wang Q, Shen WL, Liu LK (2021). Cancer-associated fibroblasts promote oral squamous cell carcinoma progression through LOX-mediated matrix stiffness. J Transl Med.

